# Production of lactic acid from pasta wastes using a biorefinery approach

**DOI:** 10.1186/s13068-022-02222-x

**Published:** 2022-11-21

**Authors:** Cristina Marzo-Gago, Joachim Venus, José Pablo López-Gómez

**Affiliations:** 1 Microbiome Biotechnology Department, Leibniz Institute for Agricultural Engineering and Bioeconomy, Max-Eyth-Allee 100, Potsdam, Germany; 2 Department of Chemical Engineering and Food Technology, Faculty of Sciences, University of Cádiz, Pol. Río San Pedro S/N, Puerto Real, 11510 Cádiz, Spain; 3 National Center for Biotechnological Innovations of Costa Rica (CENIBiot), 1174-1200 San José, Costa Rica

**Keywords:** Wheat bran, Pasta waste, Solid-state fermentation, *Bacillus coagulans*, Pilot scale, Enzymatic hydrolysis, Food waste

## Abstract

A total of 398 kt of pasta waste (PW), generated during the production process of pasta, were produced in 2021. Due to its chemical composition and practically zero cost, PW has already been studied as a raw material for the production of lactic acid (LA) through fermentations. The main objective of this article was to improve the economic viability of the process by replacing commercial enzymes, necessary for starch hydrolysis in PW, with raw enzymes also produced from wastes. Enzyme synthesis was achieved through solid-state fermentation (SsF) of wheat bran by *Aspergillus awamori* or *Aspergillus oryzae* at various moisture contents. The maximum amylase activity (52 U/g dry solid) was achieved after 2 days of fermentation with *A. awamori* at 60% of moisture content. After that, the enzymes were used to hydrolyse PW, reaching 76 g/L of total sugars, 65 g/L of glucose and a yield of 0.72 g_glu_/g_ds_ with the enzymes produced by *A. awamori*. Subsequently, the hydrolysate was fermented into LA using *Bacillus coagulans* A559, yielding 52 g/L and 49 g/L with and without yeast extract, respectively. Remarkably, compared to the process with commercial enzymes, a higher LA yield was reached when enzymes produced by SsF were added (0.80 g_LA_/g_glu_). Furthermore, the productivities between the two processes were similar (around 3.9 g/L/h) which highlights that yeast extract is not necessary when using enzymes produced by SsF.

## Introduction

The reduction of food waste has become an important target of the European Commission by promoting a system for sustainable food production [1]. One of the main objectives is building a system in which food losses at the production stage, where 30% of the total food waste is generated, are limited. Wastes from the food production stage can be used as raw materials to obtain value-added products following environmentally friendly processes. As residues, they are low-cost materials and do not compete with the food chain. Additional advantages of using this type of waste are that they have a homogeneous composition, a high concentration of carbohydrates and are produced in large quantities [2].

Pasta is massively consumed all over the world, with a total of 16.9 Mt produced in 2021 [3]. In the same year, the country with the highest consumption of pasta was Italy, where 23.5 kg of pasta was consumed per person per year [3]. However, the production of such amount of pasta also generates an important amount of food waste. In the study performed by Principato et al., food losses and wastes were estimated in 1979 g per kg of pasta manufactured [4]. Nearly 1.2% of this food losses and wastes were pasta wastes obtained during the production process (PW). This implies that around 398 kt of PW were generated in the whole world in 2021.

The valorisation of this residue, PW, can be performed via a sequential hydrolysis and fermentation process. The high starch content (60% w/w) of PW means that it can be enzymatically hydrolysed by the addition of amylases in a process which requires low energy consumption and that runs under mild and non-corrosive conditions [5]. However, a downside of the hydrolysis is that it requires expensive enzymes produced mainly from pure sugars. It is well known that the use of such costly enzymes increases the costs of the processes and hinders their implementation at an industrial scale. Alternatively, enzymes can be produced by fermentation of agricultural wastes. Many studies are available in the literature where enzymes produced via solid-state fermentation (SsF) were successfully employed in the hydrolysis of organic wastes such as sugarcane bagasse [6], sugar beet pulp [7], waste bread [8], organic fraction of municipal solid waste (OFMSW) [9, 10], etc.

SsF is a process where microorganism grows in an environment without free water [11]. A solid material is used in these fermentations that could be used as nutrient source or as supporting material infused with nutrients essential for microbial growth [12]. SsF is standard and steady developing bioprocess to produce enzymes [13]. The production of amylases by SsF has been investigated on different industrial wastes, nonetheless, it has been consistently reported that wheat bran (WB) is one of the best substrates to produce amylases in SsF by *Aspergillus* species [14]. Furthermore, Kunamneni et al. tested several substrates for the production of this enzyme with *Thermomyces lanuginosus*, founding that WB was also the one that produce higher enzyme activity [15].

Lactic acid (LA) is a valuable platform chemical with wide-ranging applications [16]. It is commonly used in the food, pharmaceutical, cosmetic, and textile industries [17]. For instance, it is used as preservative and pH adjusting agent in the food industry. Recently, LA has also gained interest as a precursor of biodegradable polymer (PLA). Due to the wide variety of applications, the market value of LA raised to 2.7 billion dollars in 2020 and is predicted to grow at an annual rate of 8.0% from 2021 to 2028 [16]. Although LA can be produced via chemical processes, the fermentative one is preferred because it produces pure optical enantiomers. However, in most cases the fermentation media require the supplementation of nutrients to reach higher yields and good microbial growth. Many studies supplement the medium with yeast extract (YE), which raises costs. This is a significant drawback when the process is to be implemented on an industrial scale [18].

In this article, the enzyme production by solid-state fermentation was incorporated to the lactic acid production process from pasta waste following a biorefinery approach. The main objective of this research was to improve the production process of LA from PW, making the process more economically viable. To achieve it, the addition of enzymes produced by SsF was evaluated as a substitute of the commercial enzymatic cocktail. As previously stated, the enzymes cost is the main bottleneck of the enzymatic hydrolysis process. WB was selected as the best raw material, based on the literature, for amylase production under SsF, and two fungi (*Aspergillus awamori* and *Aspergillus oryzae)* were studied at various moisture contents and fermentation times. Finally, LA production was compared on the different hydrolysates produced, testing also the supplementation of YE in the fermentation. Additionally, the price of LA was calculated based only on the enzymes and supplements studied in order to see the economical differences between the different methodologies applied.

## Materials and methods

### Substrate

WB was used in SsF to produce enzymes. The material was provided by a farm (SC ALBATROS SRL) in Romania and was kept at – 25 °C until use. Prior to SsF, the required amount of WB for SsF was sterilised at 121 °C for 15 min.

PW was used as the substrate during the enzymatic hydrolysis to produce sugars. It was provided as lasagne sheets of different sizes from a company in Belgium. The composition of PW was analysed in our previous work [19]. To have a homogeneous substrate at the desired concentration of PW, it was firstly mixed with a small amount of distilled water into a mixer. After that, water was added to achieve the desired PW concentration. Finally, the substrate was inactivated just before the hydrolysis by heating the medium up to 80 °C for 30 min inside the reactor [19].

### Microorganisms

Two fungal strains were used in the SsF of WB: *Aspergillus awamori* (DSM 63272) and *Aspergillus oryzae* (DSM 1862). All the strains were stored at − 80 °C and were reactivated in potato dextrose medium at 30 °C for 5 days. After that, the spores collected from the liquid medium were seeded in Petri dishes with potato dextrose agar and incubated at 30 °C for 5 days. Then, the spores were collected with a solution of NaCl (0.9% w/v) and the concentration of spores was calculated using a Thoma cell counting chamber.

*Bacillus coagulans* A559 was used in LA fermentations. This strain belongs to the strain library of the Microbiome Biotechnology Department of the Leibniz Institute for Agricultural Engineering and Bioeconomy (Potsdam, Germany). The strain was stored at – 80 °C and was reactivated in MRS broth at 52 °C. Then, the strain was grown in MRS agar slants at 52 °C. After that, the whole content of the slant was transferred into an Erlenmeyer flask with 60 mL of MRS. To stabilise the pH during the growth of the microorganism, the MRS medium was supplemented with 0.7 g EVERZIT^®^ Dol 0.5–2.5 mm (Evers, Germany), composed mainly of CaCO_3_ (68%), MgO (25%), CaO (1%) and MgCO_3_ (5.6%). The flask was incubated at 40 °C and 100 rpm for 16 h.

### Solid-state fermentation of wheat bran

The SsF of WB was performed in 300-mL Erlenmeyer flasks. The sterile solids [10 g of dry solid (g_ds_)] were added to the flask and the moisture content was adjusted to 60, 70 or 80% w/w by the addition of sterile distilled water. Also, the required volume of a spore suspension to have a concentration of 1·10^7^ spores per gramme of solid was added to the flask. The flasks were closed with a cotton plug and incubated in static conditions at 30 °C for 7 days. The SsF was performed in duplicate and, every 24 h, the whole content of the two flasks were collected for sample analysis.

### Enzyme extraction

After the SsF, enzymes were extracted by the addition of a Tween 80 solution (0.1% w/v) in a solid-to-liquid ratio of 1:8 to the Erlenmeyer flask. The flasks were placed in an incubator at room temperature for 30 min at 150 rpm. After that, the mixture was centrifuged at 5000*g* for 15 min at 7 °C to separate the solids. The supernatant was collected and stored until further analysis.

### Enzymatic hydrolysis of pasta waste

The enzymatic hydrolysis of PW was carried out with commercial enzymes and with the enzymes produced by SsF. For the first case, the commercial enzyme cocktail Stargen™ 002 was used, adding 1.28 μL/g_dpw_ (grammes of dry PW) at the beginning of the hydrolysis. For the second case, fermented WB obtained after the SsF by *A. awamori* or *A. oryzae* was added at the beginning of the hydrolysis as a source of enzymes. The SsF with the fungus *A. awamori* was performed at 60% of moisture content and the solids, containing the raw enzymes, were collected after 3 days. In the fermentation with *A. oryzae*, the SsF was carried out at 70% of moisture content and the solids, containing the raw enzymes, were collected 2 days of fermentation. In both cases, SsF was performed with 10 g_ds_ of WB in a 300-mL Erlenmeyer flask closed with cotton, incubating the flask at 30 °C. Immediately after SsF, the entire flask contents were added to the reactor to start the enzymatic hydrolysis of PW.

The hydrolysis was carried out in a 2-L reactor with a working volume of 1 L. The temperature and the stirrer speed were set at 50 °C and 300 rpm, respectively. Before the hydrolysis, the empty reactor was autoclaved at 121 °C for 15 min. After that, 200 g of PW were blended with 1 L of distilled water and were added to the reactor. Then, the reactor (containing the PW) was heated up to 80 °C for 30 min to inactivate the media [19]. After that, the media was cooled down until 50 °C. Afterwards, the pH was adjusted to 5 at the beginning of the hydrolysis, but was not regulated after that. Finally, enzymes were added to the reactor to start the enzymatic hydrolysis.

Samples were withdrawn periodically, inactivated at 95 °C for 20 min and kept at − 20 °C until further analysis. The enzymatic hydrolysis was performed in duplicate. Hydrolysis yields were calculated as the mass of sugars obtained (glucose or total sugars) divided by the initial mass of dry solids (g_glu_/g_ds_ or g_TS_/g_ds_), considering the initial dry weight of PW and WB when fermented WB was added to the hydrolysis as enzyme source.

### Lactic acid fermentation of pasta waste hydrolysate

After the enzymatic hydrolysis, the hydrolysate was centrifuged (5000*g*—15 min) and the supernatant was fermented to LA with the strain *Bacillus coagulans* A559. The fermentations were carried out at 52 °C and 200 rpm in an Eloferm multifermentation system (Biotronix GmbH, Germany) with 300 mL working volume. The bioreactors were inoculated with 5% v/v of the preculture grown as detailed in Section “Microorganisms” and the pH was controlled at 6.0 with a NaOH solution (20% w/v).

Samples were withdrawn periodically, inactivated at 95 °C for 20 min and kept at − 20 °C until further analysis of sugars and LA. The LA fermentation was performed in duplicate.

### Enzymatic hydrolysis and lactic acid fermentation at pilot scale

LA production was studied at the pilot scale through sequential hydrolysis and fermentation in a 72-L BIOSTAT UD bioreactor (B-Braun Biotech, Germany) with a working volume of 50 L. PW (200 g/L) were added into the reactor and heated up at 80 °C for 30 min to inactivate the media [19]. After that, the temperature and the agitation of the bioreactor were set to 50 °C and 200 rpm, respectively. Then enzymes, at a concentration of 1.28 µL/g_dpw_ were added. After 24 h of hydrolysis, the bioreactor settings were configured to perform the LA fermentation. Thus, temperature was adjusted to 52 °C, pH was raised to 6, and the hydrolysate was supplemented with 5 g/L of yeast extract. The inoculum (A559) for the fermentation was prepared as described in Section “Microorganisms” (MRS medium) in a 2-L BIOSTAT bioreactor (Sartorius AG, Germany). During the fermentation, the pH was maintained at 6 by the addition of NaOH (20% v/v). Samples were taken regularly, inactivated at 95 °C for 20 min and kept at − 20 °C until further analysis.

The main objective of this study was to evaluate if the removal of solids prior the fermentation could improve our previous results by reducing the lag phase of the experiments. Therefore, another experiment was performed with the same conditions, but the hydrolysate was microfiltrated before the LA fermentation.

### Analytics

#### Moisture content

The moisture content was measured during the SsF. Samples (1 g) were taken before the enzyme extraction. The samples were weight in a ceramic cup and dried in an oven at 105 °C for 24 h. The moisture content was determined as the difference on weight between the wet and dried samples divided by the weight of wet sample.

#### Determination of sugars and organic acid concentration

Glucose, fructose, xylose, arabinose, LA and acetic acid were quantified by high-performance liquid chromatography (HPLC). HPLC was carried out with a Eurokat H column (300 mm × 8 mm × 10 µm; Knauer, Berlin, Germany) and a refractive index detector RI-71 (SHODEX, Tokyo, Japan). The mobile phase had a flowrate of 0.8 mL/min of 5 mM H_2_SO_4_ and the injection volume was 10 µL. LA L-/D-isomer ratio was measured by HPLC (Chiralpak^®^MA( +) column (50 mm × 4.6 mm × 3 µm; DAICEL, Tokyo, Japan) with a 2 mM CuSO_4_ mobile phase and an UV detector.

#### Enzyme activity determination

Amylase activity was measured by mixing 0.5 mL of potato starch suspension (1.0% w/v in distilled water, pH 6.0) with 0.5 mL of crude enzyme extract. The mixture was incubated at 60 °C for 10 min in a water bath. Afterwards, 1 mL of 3,5-dinitrosalicylic acid (DNS) was added to stop the reaction.

After the enzyme reaction, the concentration of reducing sugars produced was determined by the DNS method. For that, the tubes containing the samples and the DNS reagent were boiled for 10 min, and then cooled down in ice water to stop the reaction. Distilled water (8 mL) was added to the tubes and the absorbance was measured at 540 nm.

The enzyme activity of each enzyme was expressed as micromole of glucose obtained per minute at the tested conditions. The assays were performed in duplicate, and the results were expressed as the average value and the standard deviation.

### Economic evaluation of lactic acid production

The production cost of lactic acid was estimated with the aim of comparing the different processes tested in this research. Even though an exhaustive estimation should be performed considering the additional equipment required for the SsF stage, a basic estimation was performed to show the influence of the enzyme cocktail and nutrient supplementation on the lactic acid price. Thus, the calculation is based on the amount of the commercial enzyme Stargen™ 002, the wheat bran or the yeast extract required to produce 1 kg of LA according to the yields obtained in each condition tested. The unit price of each component was extracted from the recent literature: enzyme Stargen™ 002 (9.26 €/kg [20]), wheat bran (0.15 €/kg [21]) and yeast extract (25.5 €/kg [22]).

### Results and discussion

In our previous study, PW was evaluated for LA production via enzymatic hydrolysis, with commercial enzymes, and submerged liquid fermentation [19]. In that work, various enzyme activities were tested for the hydrolysis of PW, and different *Bacillus coagulans* strains were screened for LA production. The separate hydrolysis and fermentation were scaled up to 50 L, reaching a yield of 0.67 g_LA_/g_dPW_.

Although good yields were previously obtained using commercial enzymes for the hydrolysis of PW, their high cost made difficult the work at the pilot scale and hindered the commercialisation of the process [23]. In fact, several authors have stated that the high cost of enzymes is a major bottleneck in the development of biorefineries based on organic wastes [24, 25]. Thus, many investigations have been carried out to produce enzymes via SsF from low-cost materials, such as agro-industrial wastes or subproducts, [26, 27]. In this work, WB was used to produce enzymes via SsF, which were then used for the hydrolysis of PW. Finally, PW hydrolysates were fermented and the results were compared to those of the process that utilised commercial enzymes for the hydrolysis.

### Enzyme production by solid-state fermentation of wheat bran

PW is mainly composed of carbohydrates, being the main component (80% w/w aprox. [28]) starch, a polymer of glucose units joined by glycosidic bonds. Therefore, PW is an interesting raw material to produce sugars by enzymatic hydrolysis, using amylase as the main enzyme. The production of amylase by SsF has been studied on several raw materials such as WB, dried potato peel, dried fruit waste, rice bran, groundnut cake, coconut cake, soybean powder, bagasse, paddy husk, to name a few [29, 30]. However, WB has shown the highest yield on amylase activity [29, 30]. Amongst others, one of the main factors influencing enzyme production is the microorganism used to ferment the solid. In this sense, two fungus strains were tested as amylase producers, *Aspergillus awamori* and *Aspergillus oryzae*. Moreover, the enzyme production was optimised by studying different fermentation times of the solid and three moisture content (60, 70 and 80%) as they are parameters with high impact on the enzyme production by SsF.

**Fig. 1:**
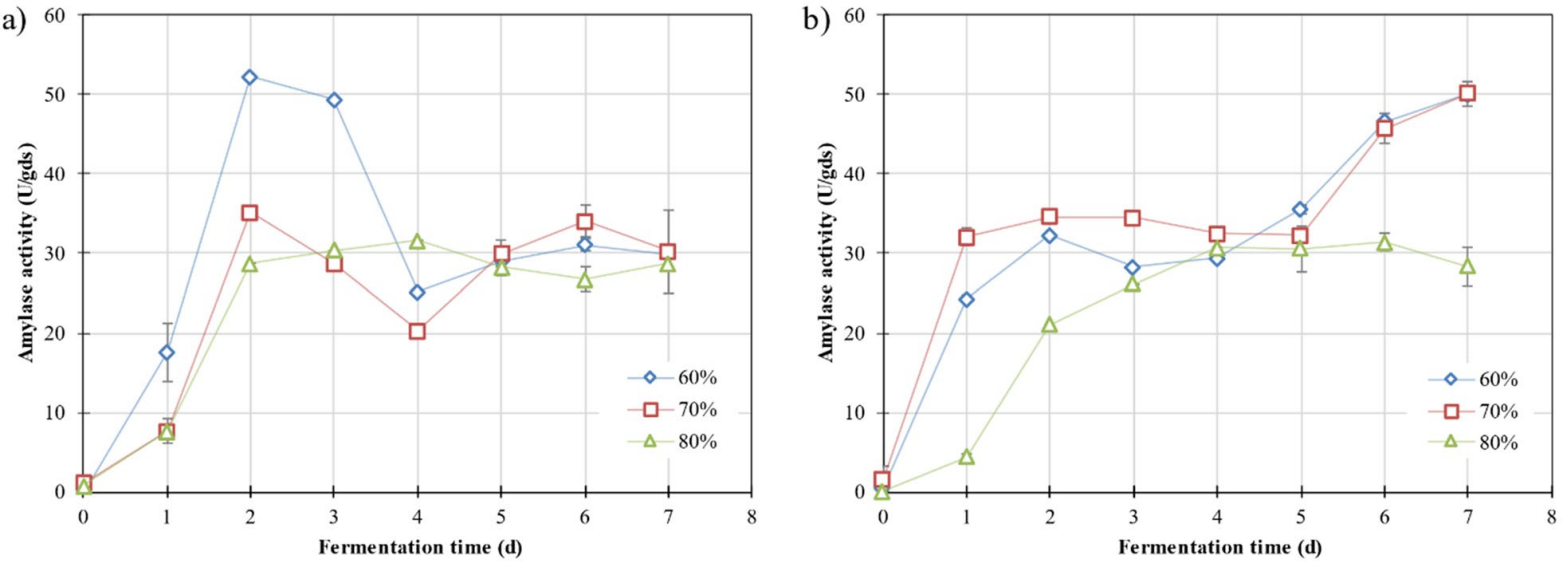
Evolution of amylase activity produced on the fermentation of WB with the fungus *A. awamori* (**a**) or *A. oryzae* (**b**) at different moisture content: 60% (blue diamond), 70% (red square) and 80% (green triangle)

The amylase production at various moisture contents with both fungi showed different trends, as shown in Fig. 1. In the fermentation carried out with *A. awamori* (Fig. 1a), the enzyme activity increased during the first 2 days and then, it decreased until reaching a stable value of 29.79 ± 1.93 U/g_ds_. Also, from the moisture contents tested, the enzyme activity increased as the moisture content decrease from 80 to 60%. Finally, the maximum amylase activity of 52.21 ± 0.04 U/g_ds_ was reached after 2 days of fermentation at 60% of moisture content. On the other hand, the moisture content which yielded the highest enzyme activity in the fermentation performed with *A. oryzae* (Fig. 1b) was 70%. In this case, over the firsts 3 days of fermentation, enzyme production rate differed amongst the moisture contents tested, nonetheless, they reached a similar value (31.81 ± 1.97 U/g_ds_) between the fourth and fifth day of fermentation. After that, an increase on the enzyme activity was observed in fermentations with an initial moisture content of 60% or 70%, reaching a value of 50.04 ± 0.07 U/g_ds_ after 7 days of fermentation.

The amylase activity obtained in this article is comparable to other studies found in the literature. Many studies can be found in which amylase is produced by SsF of WB with different microorganisms, ranging from 48 to 9421 U/g [21]. This wide difference is due to the use of different microorganism species and the establishment of diverse fermentation conditions such as the addition of mineral salts to the fermentation medium. For instance, Tanasković et al. produced the maximum amylase activity of 107 U/g on the 7th day of fermentation by using *Bacillus sp.* TMF-2, a strain isolated from dairy product (Collection of cultures of the Faculty of Technology and Metallurgy, University of Belgrade) [11]. However, Kalia et al. obtained 192 U/g of α-amylase with the fungus *Trichoderma reesei* when WB was supplemented with salt media containing NaNO_3_ (2 g/L), K_2_HPO_4_ (1 g/L), MgSO_4_·7H_2_O, (0.5 g/L), KCl (0.5 g/L), yeast extract (2.5 g/L) [29].

**Fig. 2:**
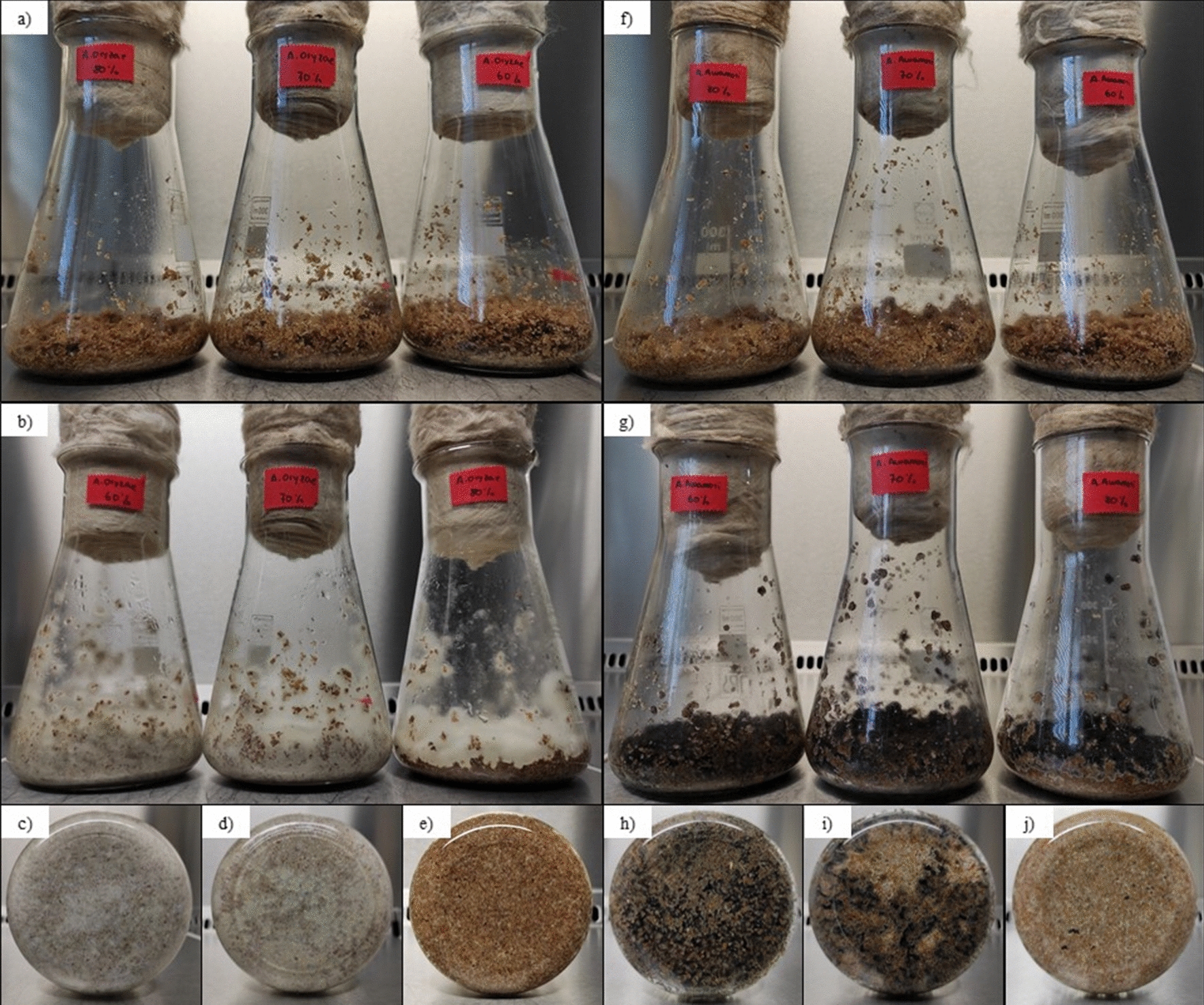
Image of *A. oryzae* before SSF at 60%, 70% and 80% moisture content (**a**), and after 3 days of SSF (**b**) at 60% (**c**), 70% (**d**) and 80% (**e**) moisture content. Image of *A. awamori* before SSF at 60%, 70% and 80% moisture content (**f**), and after 3 days of SSF (**g**) at 60% (**h**), 70% (**i**) and 80% (**j**) moisture content. Images from **c**, **d**, **e**, **h**, **i**, **j** shows the bottom of the flask

According to Kunamneni et al., the moisture content in the SsF has a great influence on the production of enzymes and their secretion into the medium as it interferes with the physical properties of the particles [15]. During the performance of the experiments reported in this article, variations on microbial growth could be observed as a result of the differences in the initial moisture content of the solids (Fig. 2). At a moisture content of 80%, the porosity of the solid was reduced and the inter-particle space was filled with water instead of air, limiting the oxygen transfer. As a result, growth was more evident on the surface of the solid particles, as seen in Fig. 2, where there is no growth on the flask walls that are in contact with the solids at 80% moisture content. However, both fungi showed uniform growth throughout the bed of solids at moisture contents of 60 and 70% (Fig. 2). Most likely, the negative effect of excess water on the growth of the microorganism was the cause for the lower enzyme activities observed at 80% moisture content.

### Enzymatic hydrolysis of pasta waste

**Fig. 3:**
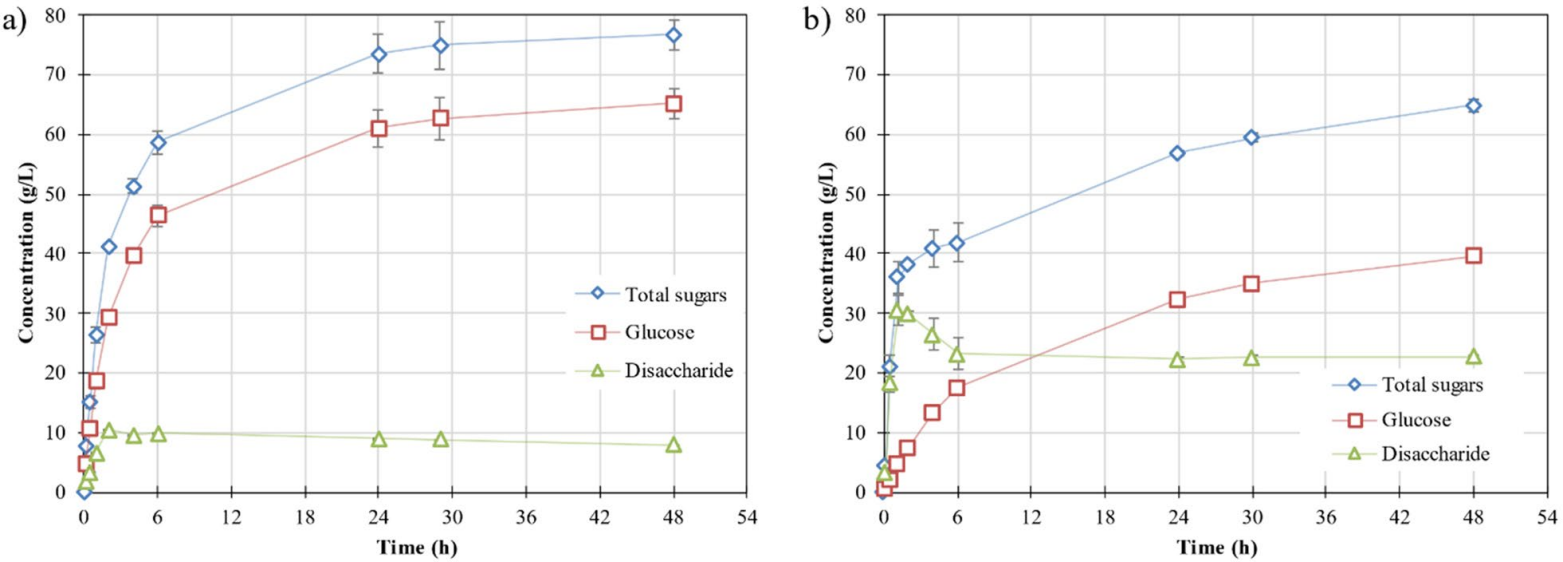
Hydrolysis of PW by adding 10 g_ds_ of WB fermented with the fungus *A. awamori* (**a**) or *A. oryzae* (**b**): concentration of total sugars (blue diamond), glucose (red square) and disaccharide (green triangle)

PW was hydrolysed with the enzymes produced by SsF. The profile for the sugars produced during the enzymatic hydrolysis of PW is shown in Fig. 3. The hydrolysis of PW by the addition of the fermented WB by *A. awamori* (10 g_ds_) produced 76.65 ± 2.59 g/L of total sugar in 48 h, 65.12 ± 2.53 g/L of which were glucose (Fig. 3a). In this way, a hydrolysis yield of 0.72 g_glu_/g_ds_ was achieved. On the other hand, the hydrolysis of PW with the addition of the fermented WB by *A. oryzae* (10 g_ds_) produced 64.84 ± 0.99 g/L of total sugars in 48 h, 39.68 ± 0.66 g/L of which were glucose (Fig. 3b). In this case, the hydrolysis yield only was 0.44 g_glu_/g_ds_.

The differences found in the amount of glucose produced are related to the concentration of disaccharides produced. For the first case (Fig. 3a), the concentration of disaccharides increased until 10.51 ± 0.26 g/L and then decreased to 7.96 ± 0.22 g/L. However, for the second case (Fig. 3b), the concentration of disaccharides reached 30.46 ± 0.43 g/L and then decreased to 22.76 ± 0.08 g/L. Such difference could be the result of a lack of an enzyme able to hydrolyse maltose produced during the hydrolysis of starch. According to the literature, the hydrolysis of starch is achieved by the synergistic action of three types of amylases: α-amylase (EC 3.2.1.1), β-amylase (EC 3.2.1.2) and γ-amylase (EC 3.2.1.3) [31]. The differences between them lie on the type of bond they hydrolyse. α-amylase is an endo-enzyme that cleaves the α-(1,4)-glycosidic linkages of amylose, releasing maltotriose and maltose, and after cleavage of α-(1,6)-glycosidic bonds of amylopectin, it releases glucose [32]. β-Amylase is an exo-enzyme that hydrolyses α-(1,4)-glycosidic bonds from the non-reducing end of the polysaccharide chain, producing maltose units [32]. γ-Amylase is an enzyme that cleaves the last α-(1,4)-glycosidic bond at the non-reducing end as well as the α-(1,6)-glycosidic linkages of the amylose and amylopectin polymers, producing glucose [32]. According to Sahnoun et al., they identified three different amylases produced by *A. oryzae* (S2) under SsF. One of them was only produced on the SsF and hydrolysed starch to maltose, maltotriose, and other maltooligosaccharides of higher polymerisation degrees, while the others two amylases were also secreted on submerged fermentation [33]. Knowing this, it seems that amylases produced by *A. oryzae* had a bigger proportion of β-amylase due to the high content of maltose on the hydrolysate which was not further hydrolysed to glucose. Meanwhile, the amylase produced by *A. awamori* could have a higher proportion of α-amylase or γ-amylase.

### Lactic acid fermentation of pasta waste hydrolysates obtained with commercial enzymes

**Fig. 4:**
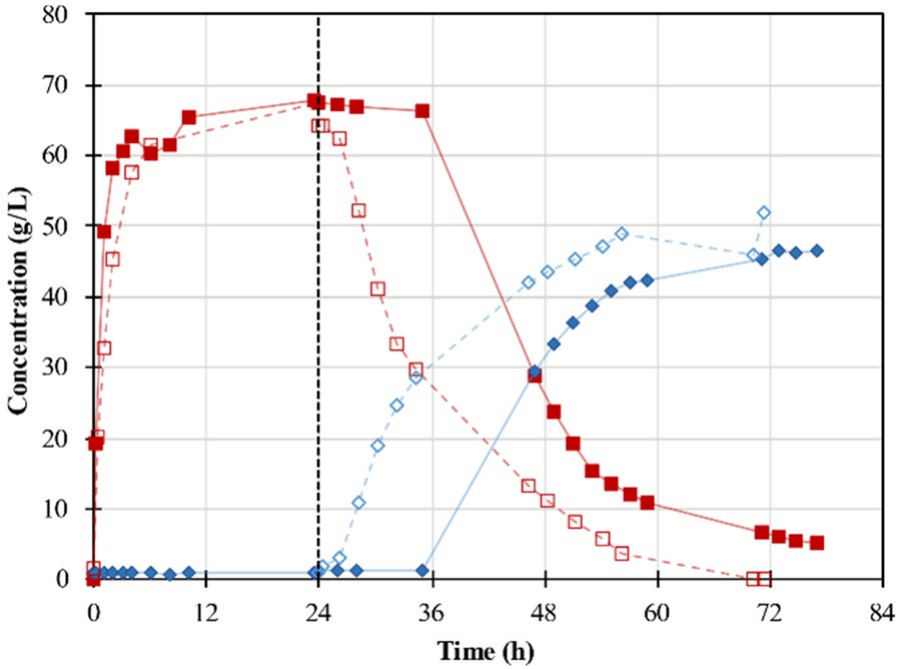
Evolution of the concentrations of total sugars (red lines) and LA (blue lines) during the enzymatic hydrolysis and lactic fermentation of PW: without solid removal (red filled square, blue filled diamond) and with solid removal (red square, blue diamond). Inoculation time is shown with a line at 24 h

In a prior report [19], PW hydrolysates were used in LA fermentations, however, the processes showed a longer lag phase when the experiments were performed at the pilot scale. It is known that remaining solid particles after the hydrolysis can affect fermentation performance [19]. Thus, in this work, solid particles were removed from the medium after hydrolysis by microfiltration before the fermentation, the results are shown in Fig. 4.

Sugar production during the enzymatic hydrolysis of PW was very fast, finishing in almost 24 h with a total sugar concentration of 67 g/L. After that, the strain *Bacillus coagulans* A559 was inoculated to produce LA. The effect of the solids removal was evident, with a clear reduction of the fermentation lag phase. In fact, there was a very short lag phase (2 h) in comparison to the fermentation without solids removal which lasted almost 14 h. Although both experiments reached similar LA concentrations (around 47 g/L) and yields (about 0.65 g_LA_/g_ds_), productivities differed significantly. A productivity of 1.56 g/L/h was reached when solids were removed from the media while 0.97 g/L/h was attained in the other case, showing a difference of 20 h between both conditions. Due to these results, the solids in the hydrolysates obtained with the enzymes produced by SsF were also removed before the fermentations.

### Lactic acid fermentation of pasta waste hydrolysates obtained with the enzymes from solid-state fermentation

**Fig. 5:**
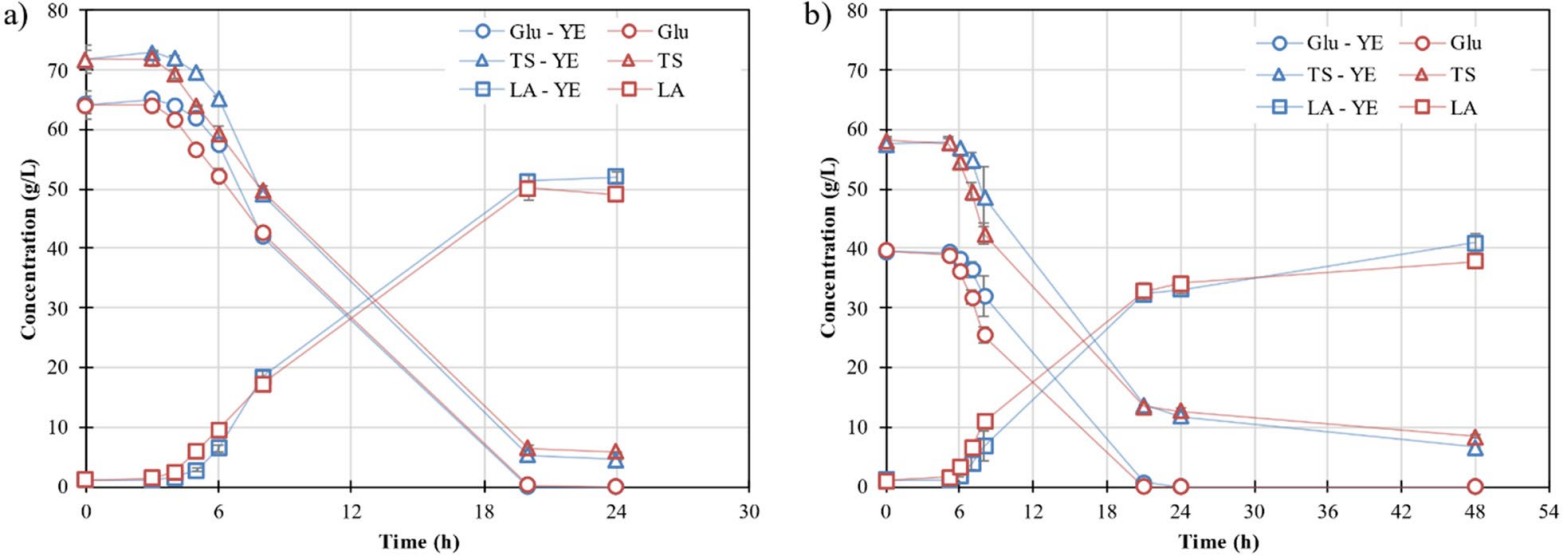
LA fermentation of the hydrolysate obtained with the enzymes produced by *A. awamori* (**a**) and *A. oryzae* (**b**): supplemented with yeast extract (glucose (blue circle), total sugars (blue triangle) and LA (blue square)) and without supplementation (glucose (red circle), total sugars (red triangle) and LA (red square))

Both hydrolysates obtained with the enzymes produced by SsF were used as media for LA production (Fig. 5). In these fermentations, the supplementation of the hydrolysate with yeast extract (5 g/L) was tested. Nevertheless, a very similar trend was observed when fermentations were carried out with and without yeast extract supplementation. This result was highly relevant given that the addition of yeast extract has a large impact on the total cost of the process. Yeast extract is expensive and many authors have searched for alternatives to substitute yeast extract for a low-cost supplement [34–36]. In our case, the addition of fungal fermented solids into the enzymatic hydrolysis seemed to provide some nutrients, being unnecessary to add other nitrogen supplementation during the fermentation.

The highest LA concentration was achieved with the hydrolysate produced with the enzymes from *A. awamori*. This was expected since the glucose concentration was higher in that hydrolysate. By the end of the fermentation, the concentration of LA obtained on the hydrolysates produced with the enzymes from *A. awamori* and *A. oryzae* were 52 g/L and 41 g/L in the fermentation with yeast extract, and 49 g/L and 38 g/L in the fermentation without yeast extract, respectively. In all cases studied, the optical purity of lactic acid was determined higher than 99% of L-lactic acid.

Some recent studies on the production of LA from agricultural or food wastes are summarised in Table 1. Between the selected studies, the maximum LA yield and productivity were attained via the continuous fermentation of rice straw. However, the yields achieved in this study were comparable to the one for corn stover and were higher than the ones for the organic fraction of municipal solid waste (OFMSW) and sugar beet pulp. Additionally, the productivity was higher on the present study, except for the continuous fermentation.

**Table 1:** Recent published works on LA production from agricultural or food waste

Carbon source	Nitrogen source	Microorganism	Fermentation mode	LA			References
				Conc. (g/L)	P (g/L/h)	Y (g/g)	
Pasta waste	–	*Bacillus coagulans* A559	SHF	49	3.87	0.61	This study
Pasta waste	YE	*Bacillus coagulans* A559	SHF	52	6.01	0.65	This study
Rice straw	YE, VB	*L. delbrueckii subsp. delbrueckii* NBRC 3202	MICF (continuous)	47	18.56	0.92	[36]
Corn stover	YE, PEP, BE	*P. acidilactici* ZY15-ΔackA2::CGS9114_RS09725	SSCF (Batch)	115	1.60	0.61	[37]
Cheese whey powder	CWP Protein, YE	*Lactobacillus bulgaricus* CGMCC 1.6970	Fed-batch	113	2.36	–	[38]
OFMSW	YE	*Bacillus coagulans* A166	SHF	60	–	0.22	[39]
Pasta waste	YE	*Bacillus coagulans* A559	SHF	48	3.43	0.67	[19]
Sugar beet pulp	YE	*Lactobacillus plantarum* CECT 748	SHF	50	0.55	0.50	[40]

YE, yeast extract; VB, vitamins B; PEP, peptone; BE, beef extract; CWP, cheese whey powder; SHF, separate hydrolysis and fermentation; MICF, membrane integrated continuous fermentation; SSCF, simultaneous saccharification and co-fermentation; Conc, concentration; P, productivity; Y, yield

### Comparison of both methodologies for lactic acid production

Both processes studied for LA production, with commercial enzymes and with enzymes produced by SsF, were evaluated based on LA productivity and yields estimated from the enzymatic hydrolysis and the LA fermentation steps (Table 2). Considering the enzymatic hydrolysis step, similar yields (Y g_glu/_g_ds_) were reached when fermented WB by *A. awamori* and the commercial enzyme cocktail Stargen™ 002 were used to hydrolyse PW (0.72 and 0.79 g_glu_/g_ds_). However, a lower yield was obtained when fermented WB by *A. oryzae* was added (0.44 g_glu_/g_ds_). Nevertheless, the yields of total sugars from dry solids (Y g_TS/_g_ds_) were higher when the enzymes produced by SsF were used, reaching 0.85, 0.72 and 0.82 g_TS_/g_ds_ with the enzymes produced by *A. awamori*, *A. oryzae*, and with Stargen™ 002, respectively. It is important to note that the yields obtained in the hydrolysis carried out with the fermented solid contained, in addition to PW, 10 g_ds_/L of WB, which can be hydrolysed with the enzymes produced during SsF. Moreover, the difference found between *A. awamori*’s enzymes and the commercial one could be the result from the production of a wide and more robust variety of enzymes. Several authors have reported the production of cellulases, xylanases or pectinases by *A. awamori* on SsF [27, 41], which may hydrolyse the non-starch material of PW. Likewise, the differences between *A. oryzae*’s enzymes and the others were the result of the non-complete hydrolysis of maltose to glucose.

**Table 2:** Yields of hydrolysis (g_glu_/g_ds_), LA fermentation (g_LA_/g_ds_) and LA productivity (g/L/h) with the addition of yeast extract (YE) and without supplementation (w/o)

Experiment	g_glu_/g_ds_	g_TS_/g_ds_	g_LA_/g_glu_ (YE)	g_LA_/g_glu_ (w/o)	g_LA_/g_ds_ (YE)	g_LA_/g_ds_ (w/o)	LA (g/L/h) (YE)	LA (g/L/h) (w/o)
*A. awamori*	0.72	0.85	0.80	0.75	0.58	0.55	6.01	3.87
*A. oryzae*	0.44	0.72	0.63	0.58	0.46	0.42	4.04	4.48
Stargen™ 002	0.80	0.82	0.76	–	0.61	–	3.88	–

Regarding the LA fermentation step (Table 2), the highest yield of LA from glucose (Y g_LA/_g_glu_) and from dry solids (Y g_LA/_g_ds_) were achieved with the hydrolysate obtained with the enzymes produced by *A. awamori* supplemented with yeast extract (0.80 g_LA_/g_glu_ and 0.58 g_LA_/g_ds_). Moreover, a similar yield (0.75 g_LA_/g_glu_) was obtained in the experiment using commercial enzymes supplemented with YE and in the experiment using the enzymes from *A. awamori* without YE supplementation. Thus, the same amount of LA obtained in experiments with the addition of a commercial enzyme cocktail and yeast extract can be obtained using only the enzymes produced by *A. awamori* in SsF. The same effect was observed regarding LA productivity, the highest productivity was achieved with the enzymes from *A. awamori* supplemented with YE, while a similar productivity was reached between the crude enzymes without YE supplementation and the commercial enzymes with YE supplementation.

Besides the high yield attained, the production cost of LA is also important in the economic viability of the process at industrial scale. For this reason, the production cost was estimated based on the cost of WB, enzymes and yeast extract supplements. Although an exhaustive economic study should be required to confirm the viability of the process, some differences were observed taking in consideration only these factors. The cost of the enzymes and supplements to produce 1 kg of LA according to the process followed are summarised in Table 3. As clearly seen, the addition of YE and the commercial enzymes are the factors with a higher impact on the final cost of LA. Therefore, the production of lactic with the commercial enzyme Stargen™ 002 and with the YE supplementation was 12 times higher than the one with only the addition of fermented WB by *A. awamori.*

**Table 3:** Cost comparison of LA production considering the enzymes produced by SsF (*awamori, oryzae*), the commercial enzyme cocktail (Stargen™ 002) and the supplementation of the LA with yeast extract (+ YE)

	*A. awamori* + YE	*A. awamori*	*A. oryzae* + YE	*A. oryzae*	Stargen 002 + YE
Enzyme	– €	– €	– €	– €	0.05 €
WB	0.03 €	0.03 €	0.04 €	0.04 €	– €
YE	2.45 €	– €	3.11 €	– €	2.62 €
€/kg LA	2.48 €	0.03 €	3.15 €	0.04 €	2.68 €

## Conclusions

The commercial enzyme cocktail Stargen™ 002, used to hydrolyse PW, was successfully replaced by enzymes produced via SsF. *A. awamori* yielded the highest amylase activity after SsF of WB for two days at 60% of moisture content. Also, higher yields were obtained during the enzymatic hydrolysis of PW when these enzymes were added. Regarding LA production, similar yields were reached between the hydrolysate obtained after the addition of the fermented WB by *A. awamori* and the one produced with commercial enzymes and supplemented with YE. To conclude, the production of LA from PW was improved by the addition of enzymes produced through solid-state fermentation, with no addition of yeast extract during lactic fermentation.

## Data Availability

Not applicable.

## Abbreviations

BE: Beef extract
Conc: Concentration
CWP: Cheese whey powder
DNS: 3,5-Dinitrosalicylic acid
glu: Glucose
ds: Grammes of dry solid
dPW: Dry pasta waste
LA: Lactic acid
MICF: Membrane integrated continuous fermentation
OFMSW: Organic fraction of municipal solid waste
P: Productivity
PEP: Peptone
PW: Pasta waste
SHF: Separate hydrolysis and fermentation
SSCF: Simultaneous saccharification and fermentation
SsF: Solid-state fermentation
TS: Total sugars
VB: Vitamins B
WB: Wheat bran
w/o: Without supplementation
Y: Yield
YE: Yeast extract
